# A ‘Real-Life’ Experience on Automated Digital Image Analysis of FGFR2 Immunohistochemistry in Breast Cancer

**DOI:** 10.3390/diagnostics10121060

**Published:** 2020-12-07

**Authors:** Marcin Braun, Dominika Piasecka, Mateusz Bobrowski, Radzislaw Kordek, Rafal Sadej, Hanna M. Romanska

**Affiliations:** 1 Department of Pathology, Chair of Oncology, Medical University of Lodz, 92-213 Lodz, Poland; marcin.braun@umed.lodz.pl (M.B.); dominika.piasecka@gumed.edu.pl (D.P.); radzislaw.kordek@umed.lodz.pl (R.K.); 2 Department of Molecular Enzymology and Oncology, Intercollegiate Faculty of Biotechnology, University of Gdansk and Medical University of Gdansk, 80-211 Gdansk, Poland; 3 Sysmex Polska Sp. z o.o., 02-486 Warszawa, Poland; bobrowski.mateusz@sysmex.pl

**Keywords:** machine learning algorithm, AI, CaseViewer, QuantCenter, breast cancer, FGFR2, image analysis

## Abstract

We present here an assessment of a ‘real-life’ value of automated machine learning algorithm (AI) for examination of immunohistochemistry for fibroblast growth factor receptor-2 (FGFR2) in breast cancer (BC). Expression of FGFR2 in BC (*n* = 315) measured using a certified 3DHistech CaseViewer/QuantCenter software 2.3.0. was compared to the manual pathologic assessment in digital slides (PA). Results revealed: (i) substantial interrater agreement between AI and PA for dichotomized evaluation (Cohen’s kappa = 0.61); (ii) strong correlation between AI and PA H-scores (Spearman r = 0.85, *p* < 0.001); (iii) a small constant error and a significant proportional error (Passing–Bablok regression y = 0.51 × X + 29.9, *p* < 0.001); (iv) discrepancies in H-score in cases of extreme (strongest/weakest) or heterogeneous FGFR2 expression and poor tissue quality. The time of AI was significantly longer (568 h) than that of the pathologist (32 h). This study shows that the described commercial machine learning algorithm can reliably execute a routine pathologic assessment, however, in some instances, human expertise is essential.

## 1. Introduction

Recent years have witnessed dynamic development of machine learning algorithms, broadly known as “AI”, designed for different, mostly diagnostic purposes [1]. FDA’s approval has already been granted for implementation of AI in radiology, cardiology, ophthalmology and dermatology [1,2,3,4,5].

In pathology, where histological assessment is the key to the diagnosis and decision-making for the optimal patient care, digitalization and whole slide imaging are gaining recognition as a likely solution to improve accuracy, reproducibility and efficiency of the diagnostic process. In addition to other advantages, including elimination of cumbersome pathological visualizing hardware, possibility for working-from-distance (telepathology) [1,6] and digitalizing of histopathological slides opens avenues for development of new machine learning algorithms [6,7]. Rapid progress in AI research in pathology resulted already in development of accurate tools for Gleason scoring in H&E prostate cancer biopsies [8,9], lymph node metastases recognition in H&E breast cancer specimens [10], assessment of immunohistochemical expression of HER2 in breast cancer [11] and several others [1,6,7]. Although AI holds a great promise to improve histopathological evaluation or even outperform human expertise, several drawbacks associated with high variability in sample types, histopathological techniques, quality of material and choice of diagnostic criteria [1,6,7] need to be overcome before it can be successfully implemented in routine pathological assessment. Hence, reports on hands-on experience with currently developed automated trainable pathological tools are invaluable for paving the way for their effective application in both clinical practice and research.

Pannoramic scanners along with QuantCenter and CaseViewer analysis platforms have been designed by 3DHISTECH (Sysmex) for digitalization and automated evaluation of pathological slides [12,13]. These products are internationally recognized—they received five out of seven 1st awards (in High Throughput at 20× and at 40×, Image Quality at 20× and 40× and Technical categories) at the 3rd International Scanner Contest 2016 (Berlin, Germany). In addition, unlike most AI/ML tools available on the market, the QuantCenter also holds the renowned CE-IVD certificate.

Fibroblast growth factor receptor-2 (FGFR2) is an emerging histologic marker in several cancers, including breast carcinoma (BCa) [14,15,16,17,18,19,20]. We and others have recently showed that in BCa, FGFR2 is a major transducer of signals between tumour and its microenvironment (TEM), mediating interactions between TEM and hormone receptor-dependent pathways [21,22,23,24,25,26,27,28]. This implies that an accurate and unbiased assessment of FGFR2 protein level in BCa cells is a prerequisite for its potential integration into a molecular classifier designed to improve prediction of an outcome of BCa patients [14,19,20].

The aim of the study was to contest AI with human expertise in a quantitative and qualitative analysis of immunostaining for FGFR2 in a cohort of breast cancer specimens. For this purpose, the QuantCenter/CaseViewer, deemed to be the most worthy representative of commercially available pathological software, has been put to test against two pathologists in an assessment of efficiency and reliability of analytical performance.

## 2. Materials and Methods

### 2.1. Tissue Samples and Immunohistochemistry for FGFR2

Formalin-fixed, paraffin-embedded tumoral samples of 315 invasive ductal breast carcinomas, not-otherwise specified, diagnosed according to WHO 2012/2019 Classification of Breast Tumors, was collected from the Department of Pathology, Medical University of Lodz [29,30,31]. The study was approved by the Local Research Ethics Committee (No. RNN/34/16/KE, 16 February 2016).

For immunohistochemical procedures, 5-µm sections were processed following manufacturers‘ recommendations, as reported previously [23,27]. Immunohistochemical staining (IHC) for FGFR2, whose prognostic and predictive value was demonstrated in luminal breast cancer [14,20], was conducted using a mouse monoclonal anti-FGFR2 antibody (H00002263-M01, Abnova, Taipei City, Taiwan) [22,23,24,27]. To confirm specificity of the staining, additional IHC with a mouse anti-FGFR2 antibody (Sc-6930, Santa Cruz, Dallas, TX, USA) was performed in randomly selected samples. Issues caused by intra- and interlaboratory variability of immunohistochemical stain have been minimized by involving one technician who would conduct all procedures in one laboratory using the same device under same conditions. Tissue samples of gastric adenocarcinoma and lymph node were used as positive and negative controls for IHC, respectively [27].

### 2.2. Digitalization, Manual and Automated Assessment of FGFR2 Staining

**Figure 1:**
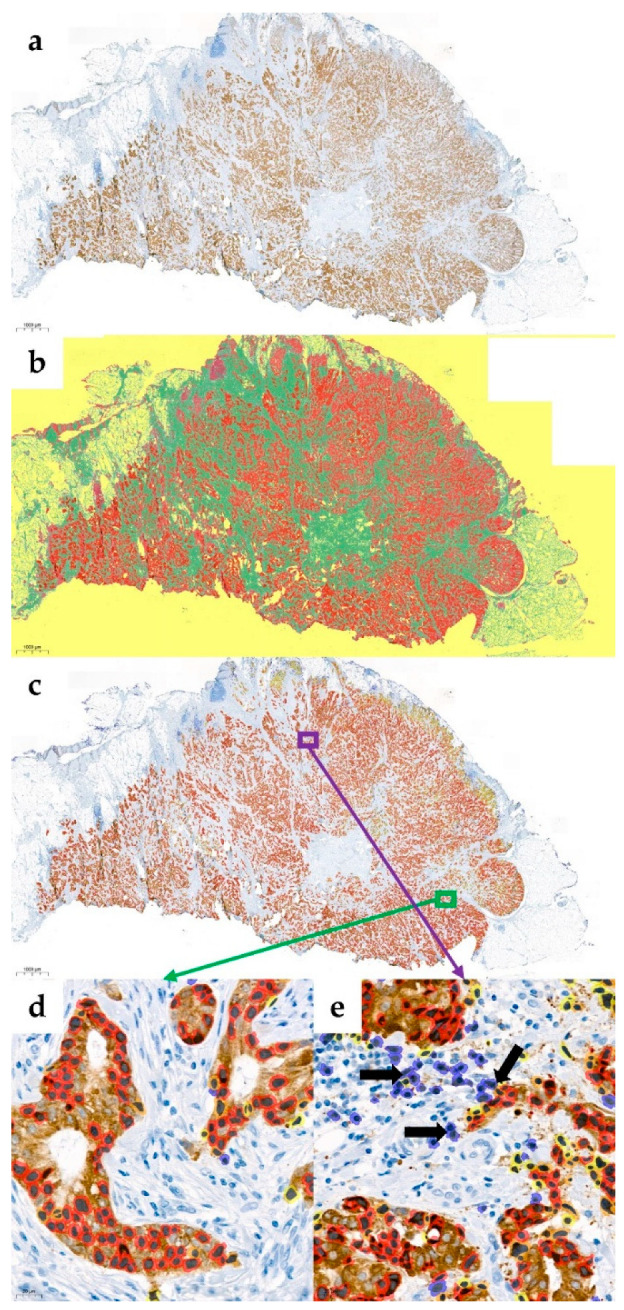
An example of automated quantification of staining for fibroblast growth factor receptor-2 (FGFR2) using H00002263-M01 antibody (Abnova, Taipei City, Taiwan) on digital scan (Pannoramic 250 Flash III scanner, CaseViewer/QuantCenter software, 3DHISTECH, Sysmex, Budapest, Hungary). The case was evaluated as strong positive for FGFR2 (FGFR2high) in both pathologic and automated scoring with H-score values of 274 (80 × 3 + 15 × 2 + 4 × 1 + 1 × 0) and 230 (59 × 3 + 18 × 2 + 17 × 1 + 6 × 0). (**a**) Immunostaining for FGFR2 with hematoxylin counterstaining, 1× magnification. (**b**) Areas/features correctly identified by the software: cancer cells (red), tumour stroma (green), and background/non-tumoral tissue (yellow), 1× magnification. (**c**) Automated measurement of FGFR2 expression under 1× magnification: red/strong—in most of the selected cancer cells, orange/moderate and yellow/weak—in small subsets of cancer cells, blue/none in several incorrectly selected non-tumoral cells. (**d**) High magnification (40×) of representative area (indicated by a green square and arrow on (c) with cancer cells correctly identified and evaluated by the software. (**e**) High magnification (40×) of representative area (indicated by a purple square and arrow on (c) with incorrectly identified cells: cancer cells overlooked by the software and false–positive tumour-associated immune cells classified as FGFR2-negative cancer cells (black arrows). Scale bars indicating 1000 µm (**a**–**c**) and 10 µm (**d**,**e**) are included in the left bottom corner of each image. Images were taken by snapshot tool integrated in CaseViewer software.

All slides were digitalized using Pannoramic 250 Flash III (3DHISTECH, Sysmex, Budapest, Hungary) and FGFR2 levels were quantified on digitalized images (MRXS file extension dedicated for CaseViewer, 3DHISTECH, version 2.3.0., Figure 1a) according to H-score approach by two independent pathologists (MB, HR). The results were presented in 0–300 scale (multiplication of percentage of positive cells by intensity of staining: 0—no staining, 1–3—increasing intensity of both cytoplasmic and membrane staining). All cases were divided into four groups (0–75, 76–150, 151–225, 226–300), and separately dichotomised into FGFR2low/high cases by 1st tercile of H-score value.

For automated quantification of staining, a two-step computational algorithm (based on the recognition of colour deconvolution) using QuantCenter software (available on 3DHistech image analysis platform, Sysmex, Budapest, Hungary) was developed. The PatternQuant module was trained for recognition of cancer cells, tumour stroma, background and non-tumoral tissue (Figure 1b). IHC Quantification tools (Nuclear-/Membrane-/Cell-/Quants) were trained for FGFR2 levels quantification in scales corresponding to pathologic evaluation (Figure 1c–e).

The QuantCenter is an embedded application for running quantification measurements on digital slides saved as MRXS files and is accessible from the CaseViewer. The image analysis was based on a developed ‘scenario’—a unique measurement profile created by linking with Quant algorithms. The main advantage of ‘the scenario’ builder is that it is possible for the user to define a unique measurement algorithm by creating a tree-hierarchical structure for the composition of measurements. The PatternQuant, which was used as the first in our algorithm, provides segmentation methods to decompose measurement area based on the pattern and intensity. Thus, in the study, the PatternQuant module was trained to specify the areas (clusters) of distinct structures. For each cluster, a unique name and colour was defined and presented in a tree-hierarchical form (Figure 1b). The above ‘Quants’ were involved to enable the automatic discrimination between cancer cells, tumour stroma, background and non-tumoral tissue. Then, we used Membrane- and Nuclear- Quants embedded in PatternQuant and the Quants were processed on areas segmented in the first step. They were then trained to measure cell morphology and stain density and to report intensity-based core ranges, overall scores and positivity percentages (including H-Score defined as above). Scoring was based on intensity-related average and deviation values modified manually by dragging the dividers between the proper score. The developed measurement procedure (algorithm) was validated on several reference slides and, after verification, was applied for automated analysis of all cases in a batch mode integrated with the software.

### 2.3. Statistical Analysis of Reliability between Automated and Expert Evaluation

Continuous data were presented as medians with interquartile ranges (IQR) and nominal data as numbers followed by percentages in brackets. For H-score (continuous variable), the interrater variability between pathologist and AI was assessed by calculation of Spearman correlation coefficients supported by Passing–Bablok regression and Bland–Altman plots [32]. For nominal variables, the interrater reliability was assessed using Cohen’s Kappa and Fleiss’ Kappa coefficients. For univariate comparisons of continuous variables between two groups Mann–Whitney U-test with Bonferroni correction for multiple comparisons was applied. Multivariate regression analysis for factors identified in the univariate analysis was conducted. The Statistica 13.0 ENG package (Dell Inc., Round Rock, TX, USA) was used and *p*-values < 0.05 were considered as statistically significant.

## 3. Results

### 3.1. Interrater Agreement between Pathologist’s and Software Assessment

**Figure 2:**
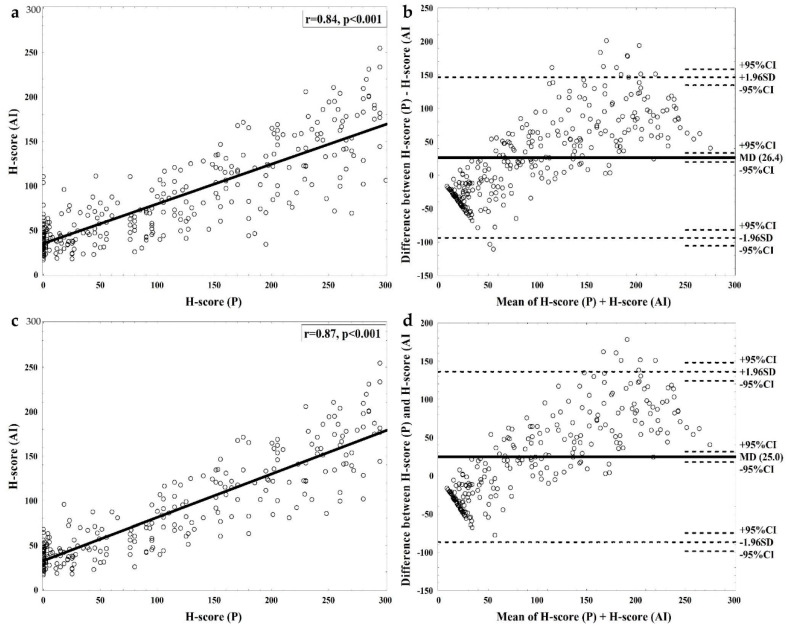
Analysis of interrater reliability of FGFR2 staining assessment. (**a**) A regression plot for correlation between automated and pathologic H-score for all cases with Spearman rank correlation coefficient and *p*-value. (**b**) A Bland–Altman plot presenting the relation between difference and mean of both H-score measurements. All cases were included. The biggest discrepancies were detected for extreme negative and extreme positive cases. (**c**) A regression plot for correlation between automated and pathologic H-score for only good quality cases with Spearman rank correlation coefficient and *p*-value. (**d**) A Bland–Altman plot presenting the relation between difference and mean of both H-score measurements. Only good quality cases were included. Fewer cases with high discordance are present and the biggest discrepancies were detected for extreme positive cases.

Median (IQR) H-score values for pathological assessment (H-score (PA)) were 95.0 (12.0–200.0) and for software-based evaluation (H-score (AI)) were 69.3 (43.2–120.2). There was a strong positive correlation between both measurements (Spearman r = 0.85, *p* < 0.001, Figure 2a). Passing–Bablok regression indicated a small constant and significant proportional error between both methods (y = 0.51 × X + 29.9, *p* < 0.001). As shown on the Bland–Altman plot, interrater variability was highest for cases with very high and very low H-score (Figure 2b). The interrater reliability for allocation of cases into four groups regarding FGFR2 intensity was moderate (Cohen’s kappa = 0.41 and Fleiss’ kappa = 0.41), while allocation of cases into FGFR2 low and FGFR2 high subgroups was of substantial interrater reliability (Cohen’s kappa = 0.61 and Fleiss’ kappa = 0.61). Figure 1 presents a case of good concordance between (PA) and (AI) evaluation (H-score of 274 and 230, strong positive, in (PA) and by (AI) examination, respectively), with areas of discrepancies between (PA) and (AI) measurement.

### 3.2. Sources of Discrepancy between Pathologist’s and Software Assessment

The possible sources for discordance between (PA) and (AI) scoring were ascertained by comparing differences of H-score regarding: (i) staining and sample quality (poor/good); (ii) tumour infiltrating immune cells (TIC) abundance (high/low); (iii) cellularity (tumour-to-stroma cells ratio—high/low); (iv) presence of necrosis (yes/no); (v) tumour heterogeneity including abundance of DCIS component and adjacent glandular healthy tissue (high/low). The univariate comparisons revealed significantly higher differences in H-score for samples with high vs. low tissue heterogeneity (57.1 (30.8–105.6) vs. 42.6 (22.1–64.7); corrected *p* = 0.049) and samples of poor vs. good quality (63.5 (34.3–105.6) vs. 40.0 (21.5–62.5); corrected *p* = 0.001), whilst no significant differences were found regarding necrosis, TIC, DCIS and cellularity (not-corrected *p* > 0.05 for all comparisons). These findings were confirmed in the multivariate analysis (β-parameter ± SE = −8.5 ± 3.5, *p* = 0.017 for low heterogeneity (high as reference), and β-parameter ± SE = −12.4 ± 2.9, *p* < 0.001 for good quality (poor as reference). The interrater agreement analyses were conducted separately for good quality slides (*n* = 262 (83.2%)). H-score (PA) in this subgroup correlated strongly with H-score (AI) (r = 0.87, *p* < 0.001, Figure 2c), and highest variability was noted for negative and very strongly positive cases (Figure 2d).

### 3.3. Time of Assessment

Completion of the automated analysis of all slides required about 22 days (31,680 min) of nonstop work of two dedicated PC units (Fujitsu Esprimo D558 i7-8700/8GB-RAM/1TB-SATA/256GB-SSD; the minimal hardware requirements stated by the producer are: Intel 3,2 GHz i5 (Quad Core), 4GB RAM, 300MB disk space) in an air-conditioned room (recommended by the software’s producer). The same task conducted by a pathologist was accomplished in 1890 min (31.5 working hours; about 5 min per sample plus 1 min for filling out the database).

## 4. Discussion

Herein, we evaluated the usefulness of AI for pathological assessment in a ‘real-life’ setting. We selected a certified commercial software for a fully automated examination of a single immunohistochemical staining in breast cancer tissue. The results confirm a great potential of this highly praised powerful investigative tool, but also indicate several shortcomings of AI, that still hinder its robust and economically justifiable application in a routine diagnostic setting.

The selected software is at the cutting-edge of AI in pathology [13,33,34,35]. Digital slides generated by 3DHistech are characterized by the best available on the market quality (the smallest pixels) and the QuantCenter algorithms are able to identify large portions of the associated pixels. Moreover, using the batch analysis mode, the QuantCenter ensures batch mode processing, i.e., automatic and uniform examination of multiple digital slides. The NuclearQuant and MembraneQuant have been awarded with CE-IVD certificates and are being currently employed in several clinical trials for ER, PR, HER2 [13,33,34,35]. All those assets prompted us to select 3DHistech as the best AI representative, capable to ‘compete’ with human expertise. Given subjectivity and cost of human expert evaluation, the promises of a universal, objective, robust, reproducible, rapid and cheap pathological assessment, that can be recorded and filed, are extremely appealing and, if maintained in a ‘real-life’ setting, they would be invaluable for routine tasks of painstaking IHC quantification. Moreover, as accurate, unbiased and uniform evaluation of potential new biomarkers, such as FGFR2, is a prerequisite for development of personalized therapies, AI would seem the preferred means to this end.

The software selected for the study was adjusted for a fully automatic examination of the whole high-resolution digitalized slide. The algorithm was successfully taught to recognize cancer cells, tumour stroma and adjacent non-cancerous tissue, and to measure the intensity of FGFR2 cytoplasmic and membrane staining, specifically in cancer cells. The final agreement between algorithm’s and pathologist’s scoring was significant and similar to other studies in the field [1,36,37].

The identified limitations of the algorithm involved inaccurate evaluation of FGFR2 in extreme cases (with no or very strong expression), in morphologically heterogenous tumors (with less glandular and more solid structures), and on slides of poor technical quality. Unfortunate as they may be, these tissue/processing imperfections are an inherent part of an everyday pathologist’s workload. While easy to be resolved by an experienced specialist, they become stumbling blocks for the machine, overcoming of which, paradoxically, calls for human assistance. Of note, we did not conduct a head-to-head comparison of available products on the market, which would be beyond the scope of the study. The choice of the software used was based on our previous experience with other similar products [38,39] and an extensive literature review. The 3DHistech QuantCenter was selected as a true representative, award-winning example of a ready-to-use adaptable deep-learning algorithm for quantitative and qualitative examination in pathology [13,33,34,35].

Furthermore, there is another aspect of AI to be carefully considered in terms of, hailed by its advocates [1,37], cost- and time-effectiveness. AI commonly requires High-Performance Computing, to which in the real-life setting, most pathology departments do not have access and, as revealed by our study, the time of the automated analysis by far (five-time as long) exceeds that of human expertise (excluding time for software optimization). These are the areas with an obvious call for improvements which, when achieved, would make AI a commonly affordable pathologist’s assistant.

## 5. Conclusions

This study provides further evidence for a potential use of AI in diagnostic and research pathology. The presented limitations of AI in the “real-life” setting emphasize a need for its further refinement to become a valuable investigative tool. As for the promise to fully replace specialist expertise, that often requires this unique and undefinable ‘human touch’, at present, only some, faint hope can be offered.
